# Effect of exercise and nutritional supplementation on health-related quality of life and mood in older adults: the VIVE2 randomized controlled trial

**DOI:** 10.1186/s12877-018-0976-z

**Published:** 2018-11-21

**Authors:** Åsa von Berens, Roger A. Fielding, Thomas Gustafsson, Dylan Kirn, Jonathan Laussen, Margaretha Nydahl, Kieran Reid, Thomas G. Travison, Hao Zhu, Tommy Cederholm, Afsaneh Koochek

**Affiliations:** 1 Department of Public Health and Caring Sciences/Clinical Nutrition and Metabolism, Uppsala University, Uppsala Science Park, 751 85 Uppsala, Sweden; 2 Nutrition, Exercise Physiology, and Sarcopenia Laboratory, Jean Mayer USDA Human Nutrition Research Center on Aging, Tufts University, Boston, MA USA; 3 Department of Laboratory Medicine, Karolinska Institute, Stockholm, Sweden; 4 Department of Food studies, Nutrition and Dietetics, Uppsala University, Uppsala, Sweden; 5 Harvard Medical School, Boston, MA USA; 6 Division of Gerontology, Beth Israel Deaconess Medical Center, Boston, MA USA; 7 Institute for Aging Research, Hebrew SeniorLife, Boston, MA USA

**Keywords:** Physical activity, Nutritional supplementation, Health-related quality of life, Depressive symptoms

## Abstract

**Background:**

Health-related quality of life (HRQoL) and absence of depressive symptoms are of great importance for older people, which may be achieved through lifestyle interventions, e.g., exercise and nutrition interventions. The aim of this investigation was to analyze the effects of a physical activity program in combination with protein supplementation on HRQoL and depressive symptoms in community-dwelling, mobility-limited older adults.

**Methods:**

In the Vitality, Independence, and Vigor 2 Study (VIVE2), community-dwelling men and women with an average age of 77.5 ± 5.4 years, some mobility limitations and low serum vitamin D levels (25(OH)Vit D 22.5–60 nmol/l) from two study sites (Stockholm, Sweden and Boston, USA) were randomized to receive a nutritional supplement or a placebo for 6 months. All took part in a physical activity program 2–3 times/ week. The primary outcome examined in VIVE2 was 400 M walk capacity. HRQoL was measured using the Medical Outcomes Study 36-item Short Form Health Survey (SF36), consisting of the Physical Component Summary (PCS) and Mental Component Summary (MCS), and depressive symptoms were measured using The Centre for Epidemiologic Studies Depression Scale (CES-D). In the sensitivity analyses, the sample was divided into sub-groups based on body measures and function (body mass index (BMI), appendicular lean mass index (ALMI), handgrip strength and gait speed).

**Results:**

For the whole sample, there was a significant improvement in both MCS, mean (95% CI) 2.68 (0.5, 4.9) (p 0.02), and CES-D -2.7 (− 4.5, − 0.9) (p 0.003) during the intervention, but no difference was detected between those who received the nutritional supplement and those who received the placebo. The results revealed no significant change in PCS or variation in effects across the sub-categories.

**Conclusions:**

This study demonstrates that a six-month intervention using a physical activity program had positive effects on mental status. No additional effects from nutritional supplementation were detected.

**Trial registration:**

Registered at ClinicalTrials.gov, March 2 2012, NCT01542892.

## Background

The complex relationship between physical activity, nutrition and health-related quality of life (HRQoL) in older adults has been described in the literature over the past decades [1–6]. A sedentary lifestyle is a risk factor for low quality of life and depression in older adults and, accordingly, physical activity can have positive effects on HRQoL and may protect against depressive symptoms [7–9]. Sarcopenia, i.e., age-related loss of muscle mass and strength, is a geriatric syndrome associated with increased risk of hospitalization, falls, mobility limitations and low HRQoL [10–13].

Moreover, optimal nutritional intake is a prerequisite for good quality of life in older persons because it prevents deficiencies and malnutrition, and nutritional supplementation may possibly contribute to upholding and improving physical function [3, 4, 14]. Interventions that combine physical activity and nutritional supplementation may have additional effects on muscle mass and function as well as on HRQoL and depressive symptoms [15]. It is therefore important to consider mental status when designing and evaluating interventions where physical activity and nutritional supplementation are combined. The study from which the data in this report originate is the Vitality, Independence, and Vigor in the Elderly 2 Study (VIVE2) which was primarily designed to evaluate the physical effects of a protein- and vitamin D-enriched nutritional supplement in combination with a physical activity program tailored for older adults with mobility limitations and vitamin D insufficiency. The primary outcome measured was change in 400 m walk performance (m/s). It was recently reported that the physical activity program resulted in an improved gait speed but there were no additional effects that could be attributed to nutritional supplementation [16]. Interestingly, potentially beneficial effects on muscle structure, i.e., reduced intramuscular fat and increased normal density muscle, were observed after nutritional supplementation [17].

In this report, we aim to present secondary analyses from the VIVE2 regarding mental status. The hypothesis was that interventions in the VIVE2 not only had effects on the participants’ physical function but also on their HRQoL, both in the physical and the mental domains, and on depressive symptoms. We therefore investigated the effect of a physical activity program in combination with protein and vitamin D supplementation on HRQoL and depressive symptoms in community-dwelling, mobility-limited older adults.

## Methods

### Participants

This study was conducted at two study sites, Boston, MA, USA and Stockholm, Sweden. Recruitment started in December 2011 and the trial was completed in November 2014. The process for recruitment, randomization, determination of sample size and other study-specific details are described elsewhere [18]. In brief, 149 community-dwelling men and women, with no severe illnesses, over the age of 70 years and with some limitations in mobility, i.e., Short Physical Performance Battery (SPPB) (0–12) ≤9 points [19], were included in the study. Further, eligible participants were required to have serum 25(OH)D concentrations of 22.5–60 nmol/l and they should not be regularly physically active, defined as not participating in moderate intensity activity more than 20 min per week. Exclusion criteria included acute or terminal illness, major surgery during the past 6 months, uncontrolled hypertension, severe pulmonary disease and current use of high-protein oral supplements or vitamin D supplements.

### Physical activity intervention

All 149 randomized participants were asked to attend a physical activity program, led by trained interventionists and consisting of three group sessions per week during the six-month trial. The groups did not exceed 15 participants. Each exercise session lasted approximately 60 min and included a warm-up, 30 min of aerobic exercise (walking), 20 min of strength exercises for lower extremities using ankle weights, and a cool down. Balance and flexibility exercises were also included. The participants were individually instructed by the interventionists on how to start the exercise intervention with lighter intensity and then gradually increase the intensity over the first 2–3 weeks of the intervention. The participants were also encouraged to be physically active outside of the session and the overall goal was to complete at least 150 min per week of moderate intensity physical activity. The exercise program is described in more detail elsewhere [18].

### Nutritional supplement

The participants were randomized to consume (once daily) either a placebo drink or a nutritional supplement (both of equal volume, 119 ml). The participants were instructed to consume the supplement/placebo immediately following the exercise session on the exercise intervention days and between meals on the other days. The nutritional supplement provided 150 kcal, 20 g of whey protein, 800 IU vitamin D and a combination of vitamins and minerals, as previously described [16]. The placebo drink was non-nutritively sweetened and provided 30 kcal per serving.

### Analytic samples

**Fig. 1:**
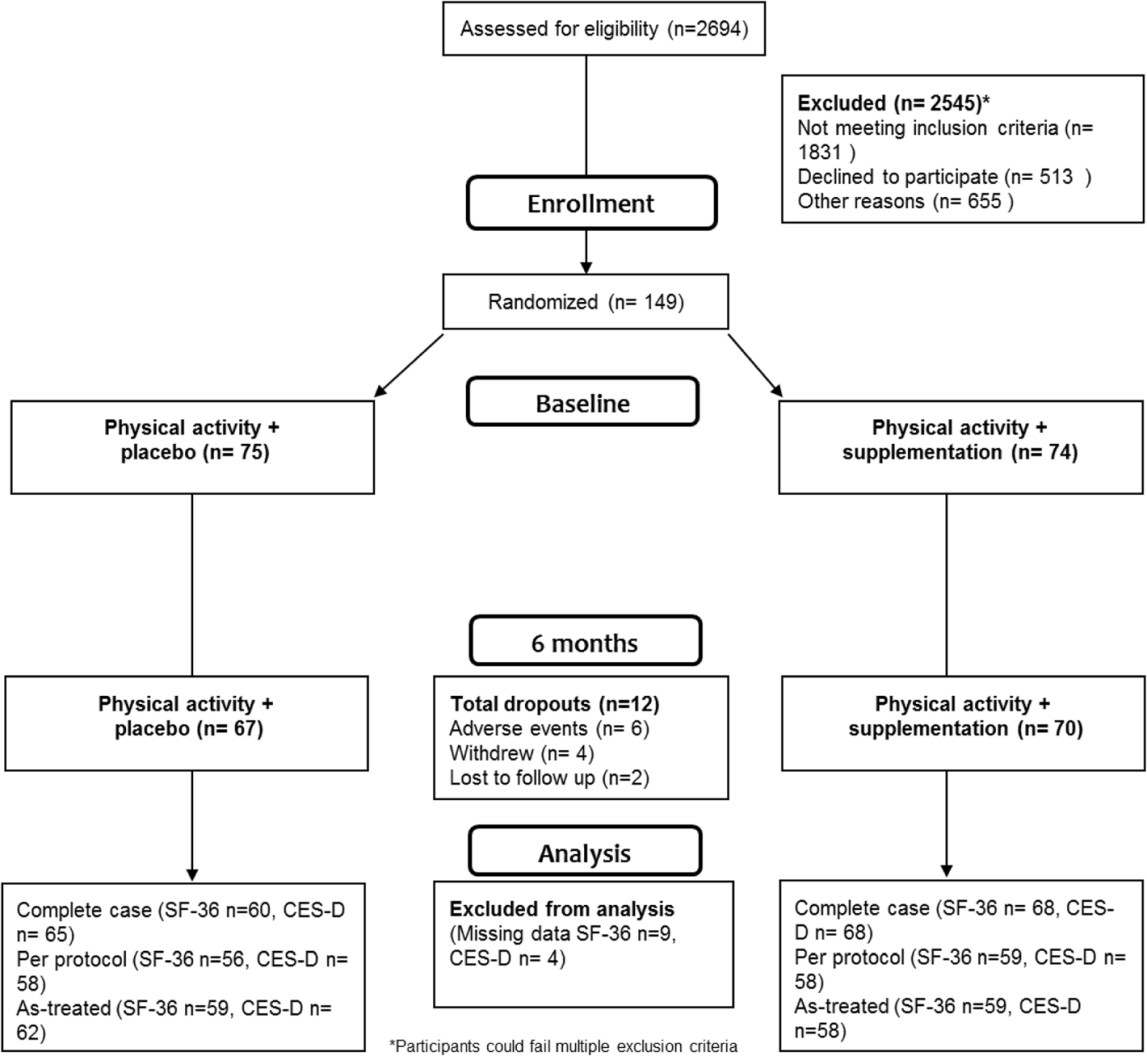
CONSORT diagraming. Flow of participants through the trial and analytical sample for this report

Of all randomized individuals (*n* = 149), 128 were included in the complete case analysis of the Medical Outcomes Study 36-Item Short Form Health Survey (SF-36) and 133 in the analysis of The Center for Epidemiologic Studies Depression Scale (CES-D) (Fig. 1). For the per-protocol analysis (PP), 120 participants were considered adherent, meaning that they attended at least 60% of the planned exercise sessions and utilized at least 60% of their doses of the nutritional supplement or placebo over the course of the six-month intervention period. In the PP-group, data were missing from the SF-36 for 5 and from the CES-D for 4 participants, respectively.

The analysis in this report also includes data on the as-treated group (*n* = 124) and complier average causal effect (CACE) estimates based on adherence data. The as-treated analysis compared data for participants by the treatment they received, irrespective of the group to which they were randomized. In the CACE estimates analysis, the effect of the randomization was maintained and compliers in the intervention group were compared with a like-for-like group in the placebo group [20, 21].

### Outcomes: health-related quality of life and depressive symptoms

Secondary outcome data from baseline and from the six-month outcome measures were used for the analyses in this paper. HRQoL was measured using the Medical Outcomes Study 36-Item Short Form Health Survey (SF-36). This tool consists of 36 items and two summary scores, a physical component summary (PCS) and a mental component summary (MCS). The SF-36 measures HRQoL in eight domains, i.e., physical function (PF), role physical (RP), bodily pain (BP), general health perception (GH), vitality (VT), social functioning (SF), role emotional (RE) and mental health (MH). The summary score ranges from 0 to 100 and higher scores indicate higher levels of HRQoL [22].

The Center for Epidemiologic Studies Depression Scale (CES-D) was used to measure symptoms of depression. The CES-D is a short, self-report measure consisting of 20 questions about symptoms experienced over the past week. The response options are scored from 0 to 3 to indicate frequency of symptoms. The score ranges from 0 to 60 with higher scores indicating more symptoms [23].

### Statistical analysis

For baseline characteristics, mean and SD or frequencies in percentage were calculated. Analysis of covariance (ANCOVA) was used to compare the outcomes on the SF-36 and CES-D between the control and intervention groups.

To complement the analyses in this paper, analysis including CACE estimates was performed using the ivreg command in STATA 13, (StataCorp. 2013. Stata Statistical Software: College Station, TX: StataCorp LP) [24]. ANCOVA was used in the sensitivity analysis where the potential effects of the intervention were compared between pre-defined subgroups based on established cut-offs for body mass index (BMI) (20–24.9 kg/m^2^, 25–29.9 kg/m^2^ and ≥ 30 kg/m^2^), appendicular muscle mass index (ALMI) (< 5.5 kg/m^2^ for women and < 7.26 kg/m^2^ for men), handgrip strength (< 20 kg for women and < 30 kg for men) and gait speed (< 0.8 m/second) [11, 25]. The sub-groups were based on baseline measures.

The models were adjusted for baseline values (MCS, PCS and CES-D), sex, age and study site. All analyses were conducted using STATA13, all statistical tests were two-sided, and differences at *p* < 0.05 were accepted as significant [24].

## Results

### Basic characteristics

As previously reported [16], the Mini Nutritional Assessment-Short Form (MNA-SF) showed a mean score of 13.3 (1.2) at baseline suggesting that the study participants had normal nutritional status. Very few (*n* = 15) were at risk of malnutrition at study start. Moreover, the mean BMI was 28.1 (3.6) and 77% were overweight or obese. The mean appendicular lean mass index (ALMI) kg/m^2^ was 7.3 (1.1). The average baseline score of the participants was 7.9 (1.2), and 12% of the participants had a score lower than 7, indicating moderate limitations (Table 1).

**Table 1:** Basic characteristics of study sample at baseline (means (SD) or counts (%)

	Control (*n* = 75)	Intervention (*n* = 74)
Age, y	76.9 (4.9)	78.1 (5.8)
Female sex, n (%)	35 (47)	34 (46)
Site - USA, n (%)	39 (52)	44 (59.5)
Body mass index (BMI), kg/m^2^	28.4 (3.9)	27.9 (3.3)
BMI > 25 kg/m^2^, n (%)	60 (80)	55 (74.3)
Appendicular lean mass index (ALMI), kg/m^2^	7.1 (1.0)	7.4 (1.1)
Short physical performance battery (SPPB)	8.0 (1.1)	7.8 (1.3)
Handgrip strength, kg	28.3 (9.7)	25.0 (8.0)
Serum 25(OH)D, nmol/l	44.25 (14.75)	49.25 (17)
Mini Nutritional Assessment-Short Form (MNA-SF)	13.4 (1)	13.2 (1.4)
Per protocol, n (%)	60 (80)	60 (82)
As-treated, n (%)	62 (83)	62 (84)

Further basic characteristics are presented in the primary outcome paper of the VIVE2 trial [16].

### Effects on HRQoL and depressive symptoms

Results at baseline and at 6 months for the PCS, MCS and CES-D are shown in Table 2. There was a significant improvement for the total sample in both MCS mean scores (2.7, 0.5; 4.9) and CES-D mean scores (− 2.7,-4.5;-0.9) during the six-month intervention, but no difference was detected between those who received the nutritional supplement and those who received the placebo drink. There was no significant change in the PCS mean.

**Table 2:** Health- related quality of life and depressive symptoms mean change

	Physical activity + placebo	Physical activity + supplementation	*p* between groups^*^
PCS			
Baseline	45.1 (8.1) (*n* = 66)	45.8 (7.6) (*n* = 73)	0.77
6 months	45.2 (9.1) (*n* = 60)	45.5 (8.1) (*n* = 68)	
Change from baseline to 6 months	0.04 (−1.9,1.9)	−0.7 (−2.3,0.9)	
MCS			
Baseline	53.1 (8.8) (n = 66)	50.4 (9.5) (n = 73)	0.14
6 months	56.1 (8.1) (n = 60)	53.1 (9.1) (n = 68)	
Change from baseline to 6 months	3 (0.5,5.5)^**^	2.3 (−0.1,4.8)	
CES-D			
Baseline	11.1 (6.6) (*n* = 67)	12.3 (7.7) (n = 67)	0.48
6 months	8.1 (7.7) (*n* = 65)	9.7 (7.7) (n = 68)	
Change from baseline to 6 months	−2.7 (−4.7,-1.22)^**^	− 2.8 (−4.5,-1.17)^**^	

Health- related quality of life (HRQoL) - physical component summary and mental component summary and the Center for Epidemiological Studies Depression Scale scores at baseline and at 6 months and mean change from baseline to 6 months (means (95% CI))

*CI* confidence interval, *PCS* physical component summary, *MCS* mental component summary, *CES-D* center for epidemiological studies depression scale

^*^The *p*-value is based on the result from the ANCOVA analysis. Adjusted for baseline value, sex, age and study site.^**^ Significant change within group (*p* < 0.05)

The results from the model of the estimated mean difference at 6 months for PCS, MCS and CES-D and the analysis for per protocol, as-treated and CACE estimates are shown in Table 3. These results did not alter the conclusions of the complete case analysis.

**Table 3:** Model– estimated mean difference between groups, per protocol, as-treated and CACE estimates analysis

	Model 1^a^	Model 2^b^
PCS		
Complete case	− 0.47 (− 2.81, 1.86) p 0.7	− 0.36 (− 2.73, 2.02) p 0.77
PP	−0.35 (−2.86, 2.15) p 0.78	− 0.26 (− 2.82, 2.29) p 0.84
As-treated	− 0.21 (− 2.68, 2.26) p 0.87	−0.13 (− 2.65, 2.39) p 0.92
CACE	− 0.27 (− 2.58, 2.04) p 0.82	−0.16 (− 2.48, 2.15) p 0. 89
MCS		
Complete case	−2.23 (− 5.13,0.66) p 0.13	−2.19 (− 5.14, 0.76) p 0.14
PP	− 2.09 (− 5.09, 0.91) p 0.17	−2.13 (− 5.18, 0.91) p 0.17
As- treated	−1.95 (− 4.91, 1.00) p 0.19	−1.96 (− 4.98, 1.03) p 0.19
CACE	− 1.89 (−4.72, 0.93) p 1.19	−1.87 (− 4.7, 0.96) p 0.19
CES-D		
Complete case	0.72 (−1.49, 2.93) p 0.52	0.79 (−1.39, 2.98) p 0.48
PP	0.22 (− 2.15, 2.58) p 0.86	0.17 (− 2.16, 2.51) p 0.88
As-treated	0.08 (− 2.22, 2.38) p 0.95	0.09 (− 2.17, 2.36) p 0.94
CACE	0.44 (−1.73, 2.62) p 0.69	0.54 (−1.58, 2.67) p 0.62

Model– estimated mean difference at 6 months between groups (95% CI) for PCS, MCS and CES-D and the analysis for per protocol, as-treated and CACE estimates

*CI* confidence interval, *PCS* physical component summary, *MCS* mental component summary, *CES-D* the Center for Epidemiologic Studies Depression Scale, *PP* per protocol, *CACE* complier average causal effect

^a^adjusted for baseline value

^b^adjustments for baseline, age, sex and study site. A minus sign indicates lower results in the intervention group

**Fig. 2:**
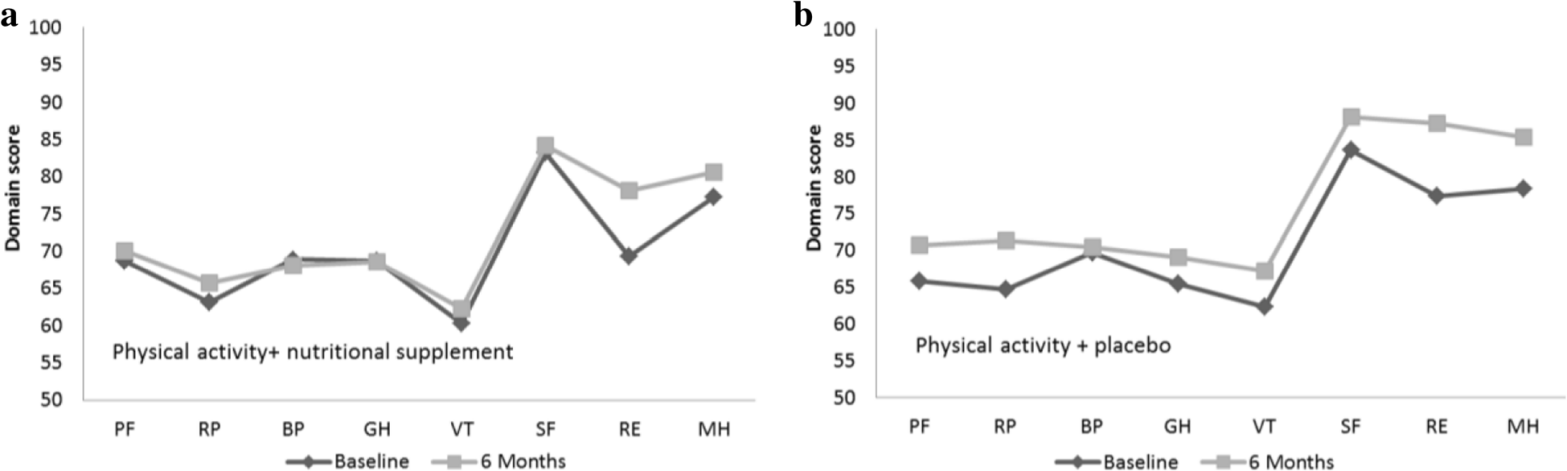
**a** and **b** Effect of the intervention on the eight domains of SF-36. Measurements at baseline and after 6 months of intervention, in the physical activity + placebo group (**a**) and in the physical activity + nutritional supplementation group (**b**). The eight domains are physical function (PF), role physical (RP), bodily pain (BP), general health (GH), vitality (VT), social function (SF), role emotional (RE) and mental health (MH)

When analyzing each of the eight domains of the SF-36 separately, improvement (in the total sample after 6 months) was detected in two domains, i.e., RE, mean (95% CI): 9.55 (2.76–16.32) (p 0.006) and MH, mean (95% CI): 5.30 (1.50–9.10) (p 0.006), with results still significant after controlling for sex, age and study site. There were no significant differences between the groups that could be attributed to the nutritional supplement (Fig. 2).

### Sensitivity analyses based on body measures and function

In a final analysis, we investigated whether sub-categories of body mass index, appendicular muscle mass index, hand grip strength and gait speed at baseline were related to the HRQOL and CES-D outcomes. These analyses revealed no variation in effects across the subcategories (data not shown).

### Missing data

In the randomized sample, data from the SF-36 were missing for 21 individuals and data from the CES-D were missing for 16 individuals. In total, data were missing for 23 individuals, *n* = 15 from the placebo group and *n* = 8 from the nutritional supplementation group. Of these individuals, 35% were adherent to the intervention (both the physical activity and nutritional supplement in the randomization) and, compared to the complete case sample, a relatively higher percentage (74%) were from the Swedish study site (p 0.003). Apart from this, basic characteristics were essentially comparable to those who completed the SF-36 and CES-D assessments (data not shown).

## Discussion

This study demonstrates that a six-month intervention using a physical activity program, supervised by trained interventionists, for mobility-limited older adults showed effects on mental health measured by the SF-36 and on depressive symptoms measured by CES-D. There were no additional effects from consuming the nutritional protein- and vitamin D-enriched supplement and the analysis for per protocol, as-treated and CACE estimates did not alter the conclusions of the complete case analysis. Furthermore, the sensitivity analyses showed no variation in effects across the subcategories based on body measures or function.

The primary outcome analyses of the VIVE2 indicated that physical exercise had a positive effect on gait speed and, in agreement with the current report, no effects were observed that could be attributed to nutritional supplementation [16]. A qualitative evaluation of the VIVE2 Swedish sample showed that the central understanding of the participants’ experiences included feelings of optimism and that social support was emphasized as being very important [26]. The positive psychological effects indicated by the qualitative analyses are possibly reflected in the results from these secondary outcome analyses, where both the mental domains of the SF-36, i.e., the MCS and mood as evaluated by the CES-D, were improved. The effects of this physical activity intervention on mental health and depressive symptoms in older adults are also consistent with results from other studies in the field [27–29]. When interpreting the results in this report, one should keep in mind that the MCS, and especially the domain mental health (MH), seems to be a good predictor of depressive disorders and that the CES-D was developed to measure depressive symptomatology and that there is, therefore, a close relationship between them.

The improvements in the MCS were driven by improvements in the SF-36 domains Role Emotional (RE) and Mental Health (MH), domains that included questions about emotional problems and time given to daily activities (RE), accomplishments (RE), nervousness (MH) and happiness (MH). These analyses of secondary outcomes did not show a difference between those who received the supplement and those who received the placebo. Previous studies have reported a potential association between vitamin D levels and HRQoL, especially in groups with various illnesses and from a short-term perspective, whereas such studies did not present evidence that long-term supplementation would affect HRQoL [30, 31]. Studies have indicated that vitamin D deficiency could be a risk factor for late-life depression, although more research is needed in this field [32, 33]. An inclusion criterion for participation in the VIVE2 was reduced serum 25(OH)D concentrations, i.e., 22.5–60 nmol/l (mean 46.8 ± 16 at baseline). The group that received the supplement increased their 25(OH)D levels an average of 36% during the intervention^16^. Thus, nutritional supplementation did not have any effect on the primary physical performance outcomes or the secondary mental outcomes. One possible reason for this absence of effect could be that, with the exception of vitamin D status, the participants were relatively well-nourished according to both the MNA-SF and BMI. It may be that the presence of more pronounced nutritional deficiencies is required for nutritional supplementation to have any measurable outcome effects, whereas there was no corresponding ceiling effect for exercise- training in this mobility-limited sample.

Interestingly, the improved gait speed that was previously reported [16] was not reflected by improvements in the PCS. In a study from 2014 aiming to try to find variables explaining HRQoL in community-dwelling older individuals, fast gait-speed was the variable that uniquely explained the variance in PCS, although the study was cross-sectional and included only 84 participants [34]. The qualitative analyses from the Swedish VIVE2 sub-sample could perhaps be of some help in interpreting these results from the PCS. In the focus groups, the participants mentioned experiencing positive physical effects from the exercise as well as some negative effects, e.g., tiredness and knee pain that could potentially have impacted HRQoL and PCS. When interpreting results from HRQoL assessments, it should also be kept in mind the potential presence of “response-shift”. Response shift occurs when individuals change their internal standards because of experiences, for example, changing their frame of reference when meeting other persons in various contexts, such as in hospital or, in this case, at a gym. Such an experience may impact their perception of quality of life [35, 36]. It can only be speculated as to whether response shifts occurred in this study, but it is possible that self-perceptions shift when changing from inactivity to a regularly active lifestyle.

It is interesting to compare the results from the VIVE2 with results from other similar studies. For example, in a RCT in older Australian women, the aim was to study the effect of combining regular progressive resistance training with either the intake of red meat (160 g cooked red meat/day, 6 days a week) or a moderate carbohydrate diet [37]. In contrast to our results, the secondary analyses showed that the combination of consuming red meat and taking exercise enhanced HRQoL when compared to the control group. This increase was driven by a better result in the PCS score, whereas no change was detected in the MCS score. Improvements in the PCS were also found in the RCT by Rondanelli et al. when investigating the effect of a tailored nutritional supplement in combination with a physical activity program in sarcopenic older adults at a geriatric hospital clinic [15]. Moreover, in a study from 2015, healthy 60-year-olds were designated to consume either a diet high in dairy protein (> 1.2 g/kg body weight/day), high in soy protein (> 1.2 g/kg body weight/day) or their “usual protein intake” (< 1.2 g/ kg body weight/day) and all participants undertook resistance training three times a week for 12 weeks. The results in this study revealed improvement in MCS with the resistance training but, as in our current study, there was no significant difference in relation to protein intake [38]. When comparing results from different studies, it is important to take variations in study design, exercise regime, supplement, and target group, into consideration.

The sensitivity analyses did not show any significant differences in results from the SF-36 or CES-D between individuals when divided into three groups based on BMI categories, BMI 20–24.9 kg/m^2^, 25–29.9 kg/m^2^ and > 30 kg/m^2^. These cut-offs were based on the traditional BMI classification of overweight and obesity for the adult population [25], whereas contemporary research indicate that BMI cut-offs for older adults may not agree with the cut-offs suggested for younger age-groups [39, 40]. Moreover, BMI does not account for changes in fat distribution or in body composition, or age-related loss of height [41, 42].

Generic instruments such as the SF-36 have some limitations. Criticisms include the instrument not being sensitive enough to detect changes and that it assumes that worsening health or physical impairment means poorer quality of life [35]. It is most likely that the reaction to health impairment might vary between individuals and mobility impairment might not automatically imply a low quality of life. Even if the SF-36 has some weaknesses, as do many instruments, it is a validated instrument that has been widely used for decades, which enables comparisons between studies.

## Conclusions

This study demonstrates that a six-month intervention using a physical activity program for mobility-limited older adults had positive effects on mental health, e.g., emotional problems, as measured by the SF-36, and on depressive symptoms as measured by the CES-D. The intervention did not have any effect on the perceived physical health (PCS) measured by the SF-36. For this reason, the results do not support the hypothesis that improved physical function obviously leads to improved self-perceived physical health. No additional effect of nutritional supplementation was detected, which may be related to the fact that the participants were fairly well-nourished. Nevertheless, the sub-group analyses showed no variation in effects across the subcategories based on body measures or function.

## Abbreviations

ALMI: Appendicular lean mass index
ANCOVA: Analysis of covariance
BMI: Body mass index
BP: Bodily pain
CACE: Complier average causal effect
CES-D: Center for Epidemiologic Studies Depression Scale
GH: General health
HRQoL: Health- related quality of life
MCS: Mental component summary
MH: Mental health
MNA-SF: Mini nutritional assessment short form
PCS: Physical component summary
PF: Physical function
PP: Per protocol
RE: Role emotional
RP: Role physical
SF: Social functioning
SF-36: Medical Outcomes Study 36-Item Short Form Health Survey
SPPB: Short physical performance battery
VIVE2: Vitality, Independence, and Vigor 2 Study
VT: Vitality
